# Supplementary material for: An ANOVA approach for statistical comparisons of brain networks
Source: Sci Rep. 2018 Mar 16;8:4746. doi: 10.1038/s41598-018-23152-5 (PMC5856783; doi:10.1038/s41598-018-23152-5)
Supplement: Supplementary file 1 — Supplementary Information [file 41598_2018_23152_MOESM1_ESM.pdf]

SUPPLEMENTARY INFORMATION OF "AN ANOVA APPROACH FOR STATISTICAL COMPARISONS OF BRAIN NETWORKS"

DANIEL FRAIMAN[1,2] & RICARDO FRAIMAN[3]

CONTENTS

1 A1- Proof of Theorem 1 3
1.1 Notation 3
1.2 A1.1- Proof i : $\mathbb{E}(T) = 0$. 3
1.3 A1.2- Proof ii : $T \to N(0,1)$. 4
1.4 A1.4- Proof iii: Under H_A 7
2 A2- Example 1 9
3 A3- HCP Resting-state fMRI functional networks 11

LIST OF FIGURES

Figure 1 Fig A1: Power of the tests as a function of the sample size for the model with parameters $\lambda_1 = 0.5$, $\lambda_2 = 2/3$, and $\lambda_3 = 0.5$. 9
Figure 2 Fig A2: **Null hypothesis.** Variance of T statistics as a function of the sample size, K for the model with parameters $\lambda_1 = 0.5$, $\lambda_2 = 0.8$, and $\lambda_3 = 0.6$. 9
Figure 3 Fig A3: Histogram of number of nodes determine by identification procedure. These results correspond to the model with parameters $\lambda_1 = 0.5$, $\lambda_2 = 0.8$, $\lambda_3 = 0.6$ and $K = 30$. 10
Figure 4 Fig A4: Relationship between variable Right Inferiortemporal Area and variable: (A) Amount of sleep , (B) Brain segmentation volume. The Spearman correlation coefficient between both variables are shown. (C) Spearman correlation matrix between the highly significant variables. 12
Figure 5 Fig A5: T–statistics as a function of (left panel) ρ and (right panel) the number of links for the variable *Picture vocabulary test*. 12

LIST OF TABLES

Table 1 Variables that partitioned the subjects in groups that present very high statistical differences between the corresponding brain networks. Only variables with $W_3 \geqslant 4$ are included. 11

Table 2 Behavioral variables that partitioned the subjects in groups that present high statistical differences between the corresponding brain networks. The W_3, W_4 and W_5 statistics are presented for different networks sizes (15 / 50 / 300). Only variables with $W_3 \geqslant 4$ are included. 11

[1] *Departamento de Matemática y Ciencias, Universidad de San Andrés, Buenos Aires, Argentina.*
[2] *Consejo Nacional de Investigaciones Científicas y Tecnológicas (CONICET), Buenos Aires, Argentina.*
[3] *Centro de Matemática, Facultad de Ciencias, Universidad de la República, Montevideo, Uruguay.*

1 A1- PROOF OF THEOREM 1

$$T := \frac{\sqrt{m}}{a}\sum_{i=1}^{m}\sqrt{n_i}\left(\frac{n_i}{n_i-1}\overline{d}_{G^i}(\mathcal{M}_i) - \frac{n}{n-1}\overline{d}_G(\mathcal{M}_i)\right) \tag{1}$$

Theorem 1. *Under the null hypothesis the statistic* T *verifies i) and ii), while* T *is sensitive to the alternative hypothesis, verifying iii).*

i) $\mathbb{E}(T) = 0$.

ii) T *is asymptotically* ($K := \min\{n_1, n_2, .., n_m\} \to \infty$) *Normal(0,1).*

iii) Under the alternative hypothesis T *will be smaller than any negative value for* K *large enough (The test is consistent).*

1.1 Notation

Let $T = \frac{\sqrt{m}}{a}Z$ with

- $Z = \sum_{i=1}^{m} W_i$
- $W_i = b_i D_{G^i}(\mathcal{M}_i) - c_i D_G(\mathcal{M}_i)$
- $b_i = \frac{\sqrt{n_i}}{n_i-1}$
- $c_i = \frac{\sqrt{n_i}}{n-1}$
- $D_{G^i}(\mathcal{M}_i) = n_i\overline{d}_{G^i}(\mathcal{M}_i)$
- $D_G(\mathcal{M}_i) = n\overline{d}_G(\mathcal{M}_i)$.

1.2 A1.1- Proof i : $\mathbb{E}(T) = 0$.

Let denote by $G_1^1, \ldots, G_{n_1}^1$ the sample networks from subpopulation 1, $G_1^2, \ldots, G_{n_2}^2$ the ones from subpopulation 2, and so on until $G_1^m, \ldots, G_{n_m}^m$ the networks from subpopulation m. Let denote without superscript $G_1, \ldots, G_n$ the complete pooled sample of networks where $n = \sum_{i=1}^{m} n_i$. And let $G_1^{k\oplus}, \ldots, G_{n_\oplus}^{k\oplus}$ be the pooled sample of networks without the sample k where $n_{k\oplus} = \sum_{h\neq k}^{m} n_h$.

The sum of the distance from the pooled sample to the average network of sample k ($\mathcal{M}_k$) can be decomposed in the following way,

$$D_G(\mathcal{M}_k) = D_{G^k}(\mathcal{M}_k) + D_{G^{k\oplus}}(\mathcal{M}_k).$$

Where

$$D_{G^k}(\mathcal{M}_k) = n_k\sum_{i<j} 2\hat{p}_k(i,j)(1-\hat{p}_k(i,j)),$$

$$D_{G^{k\oplus}}(\mathcal{M}_k) = n_{k\oplus}\Big(\sum_{i<j}\hat{p}_{k\oplus}(i,j)(1-\hat{p}_k(i,j)) + \hat{p}_k(i,j)(1-\hat{p}_{k\oplus}(i,j))\Big),$$

and $\hat{p}_k(i,j) = \frac{X_{i,j}^k}{n_k}$ is the proportion of times the link (i,j) appears in the sample k (X_{ij}^k is the number of times link (i,j) appears in sample k), and $\hat{p}_{k\oplus}(i,j)$ the proportion of times link (i,j) appears in the sample of networks $G^{k\oplus}$.

Using the fact that under H_0 it verifies that $\mathbb{E}(\hat{p}_k(i,j)) = \mathbb{E}(\hat{p}_{k\oplus}(i,j)) =: p(i,j)$, and applying the equality $\mathbb{E}\left(\hat{p}(i,j)^2\right) = p(i,j)(1-p(i,j))/n + p(i,j)^2$ it is easy to obtain that

$$\mathbb{E}(D_{G^k}(\mathcal{M}_k)) = (2n_k - 2)\sum_{i<j} p(i,j)(1-p(i,j)). \tag{2}$$

Now, since $\hat{p}_k(i,j)$ and $\hat{p}_{k\oplus}(i,j)$ are independent we obtain,

$$\mathbb{E}\left(D_{G^{k\oplus}}(\mathcal{M}_k)\right) = 2n_{k\oplus}\sum_{i<j} p(i,j)(1-p(i,j)).$$

Therefore,

$$\mathbb{E}\left(D_G(\mathcal{M}_k)\right) = (2n-2)\sum_{i<j} p(i,j)(1-p(i,j)), \qquad (3)$$

and consequently

$$\mathbb{E}\left(\frac{1}{n_k-1}D_{G^k}(\mathcal{M}_k)\right) = \mathbb{E}\left(\frac{1}{n-1}D_G(\mathcal{M}_k)\right)$$

which is the same to $\mathbb{E}\left(W_k\right) = 0$, proving that $\mathbb{E}\left(T\right) = 0$

1.3 A1.2- Proof ii : $T \to N(0,1)$.

$\overline{d}_G(\mathcal{M}_k)$ and $\overline{d}_{G^k}(\mathcal{M}_k)$ verifies the central limit because they are averages of finite variance variables. Under the Null hypothesis, both random variables have expected value zero. Then W_k has an asymptotic Normal distribution centered in zero. Moreover, $c\sum_{k=1}^{m} W_k$, where c is a non-zero constant, has an asymptotic Normal distribution centered in zero which finish the proof.

Up till now, we have shown that T is asymptotically Normal centered in zero. On the following we show that the asymptotic variance is 1.

A1.3- *The value* a

In this proof we will use only basic properties of the variance and the moments of the Binomial distribution. The value a is a sum of many simple functions. Here we calculate each of the terms of the sum.

$$Var(T) = \frac{m}{a^2}Var(Z)$$

Since we want $Var(T) = 1, \quad a = \sqrt{mVar(Z)}$

$$Var(Z) = \sum_{1\leqslant k\leqslant m} Var(W_k) + 2\sum_{1\leqslant r<t\leqslant m} Cov(W_r, W_t)$$

$$Var(W_k) = b_k^2 Var(D_{G^k}(\mathcal{M}_k)) + c_k^2 Var(D_G(\mathcal{M}_k)) - 2b_k c_k Cov(D_{G^k}(\mathcal{M}_k), D_G(\mathcal{M}_k))$$

$$\begin{aligned} Cov(W_r, W_t) = \quad & Cov(b_r D_{G^r}(\mathcal{M}_r), b_t D_{G^t}(\mathcal{M}_t)) - Cov(b_r D_{G^r}(\mathcal{M}_r), c_t D_G(\mathcal{M}_t)) \\ & + Cov(c_r D_G(\mathcal{M}_r), c_t D_G(\mathcal{M}_t)) - Cov(c_r D_G(\mathcal{M}_r), b_t D_{G^t}(\mathcal{M}_t)) \end{aligned}$$

As we have shown

$$D_{G^k}(\mathcal{M}_k) = n_k\sum_{i<j} 2\hat{p}_k(i,j)(1-\hat{p}_k(i,j)) = \frac{2}{n_k}\sum_{i<j} X_{i,j}^k(n_k - X_{i,j}^k),$$

and under H_0 is verified that $X_{1,1}^k, X_{1,2}^k, \ldots, X_{1,s}^k, X_{2,1}^k, X_{2,2}^k, \ldots, X_{s-1,s}^k$ are i.i.d. random variables with $X_{i,j}^k \sim Bin(n_k, p_{i,j})$ where s is the number of nodes in the network. And

$$D_G(\mathcal{M}_k) = D_{G^k}(\mathcal{M}_k) + D_{G^{k\oplus}}(\mathcal{M}_k) =$$

$$= \frac{2}{n_k}\sum_{i<j} X_{i,j}^k(n_k - X_{i,j}^k) + \frac{1}{n_k}\sum_{i<j}\left(n_{k\oplus}X_{i,j}^k + n_k X_{i,j}^{k\oplus} - 2X_{i,j}^{k\oplus}X_{i,j}^k\right)$$

with $X^{k\oplus}_{1,1}, \dots {}^{k\oplus}_{s-1,s}$ are iid r.v. with $X^{k\oplus}_{i,j} \sim Bin(n_{k\oplus}, p_{i,j})$ and are independent of $X^k_{i,j}$ for all i, j.
Now we calculate each of the above terms.

$\underline{Var(D_{G^k}(\mathcal{M}_k))}$

$$Var(D_{G^k}(\mathcal{M}_k)) = (\frac{2}{n_k})^2 \sum_{i<j} Var(X^k_{i,j}(n_k - X^k_{i,j})).$$

$$Var(X^k_{i,j}(n_k - X^k_{i,j})) = M_2(X^k_{i,j})n_k^2 - 2n_k M_3(X^k_{i,j}) + M_4(X^k_{i,j}) - (M_1(X^k_{i,j})n_k - M_2(X^k_{i,j}))^2,$$

where M_i is the $i-th$ moment of the Binomial Distribution.

$$\boxed{Var(D_{G^k}(\mathcal{M}_k)) = (\frac{2}{n_k})^2 \sum_{i<j} M_2(X^k_{i,j})n_k^2 - 2n_k M_3(X^k_{i,j}) + M_4(X^k_{i,j}) - (M_1(X^k_{i,j})n_k - M_2(X^k_{i,j}))^2.}$$

$\underline{Var(D_G(\mathcal{M}_k))}$

$$Var(D_G(\mathcal{M}_k)) = Var(D_{G^k}(\mathcal{M}_k) + D_{G^{k\oplus}}(\mathcal{M}_k))$$
$$Var(D_G(\mathcal{M}_k)) = Var(D_{G^k}(\mathcal{M}_k)) + Var(D_{G^{k\oplus}}(\mathcal{M}_k)) + 2Cov(D_{G^k}(\mathcal{M}_k), D_{G^{k\oplus}}(\mathcal{M}_k)) \quad (4)$$

Te second term on the right,

$$Var(D_{G^{k\oplus}}(\mathcal{M}_k)) = Var(\frac{1}{n_k} \sum_{i<j} \big(n_{k\oplus} X^k_{i,j} + n_k X^{k\oplus}_{i,j} - 2X^{k\oplus}_{i,j} X^k_{i,j})) =$$
$$= \frac{1}{n_k^2} \sum_{i<j} Var\big(n_{k\oplus} X^k_{i,j} + n_k X^{k\oplus}_{i,j} - 2X^{k\oplus}_{i,j} X^k_{i,j}\big)$$
$$= \frac{1}{n_k^2} \sum_{i<j} n_{k\oplus}^2 Var(X^k_{i,j}) + n_k^2 Var(X^{k\oplus}_{i,j}) + 4Var(X^{k\oplus}_{i,j} X^k_{i,j}) - 4n_{k\oplus} Cov(X^k_{i,j}, X^{k\oplus}_{i,j} X^k_{i,j}) +$$
$$-4n_k Cov(X^{k\oplus}_{i,j}, X^{k\oplus}_{i,j} X^k_{i,j}).$$

Each term can be expressed in a simply way in term of the moments of the binomial distribution. For example,

$$Cov(X^k_{i,j}, X^{k\oplus}_{i,j} X^k_{i,j}) = M_1(X^{k\oplus}_{i,j})(M_2(X^k_{i,j}) - M_1(X^k_{i,j})^2)$$

The third term on the right on eq. 4,

$$Cov(D_{G^k}(\mathcal{M}_k), D_{G^{k\oplus}}(\mathcal{M}_k)) = Cov(\frac{2}{n_k} \sum_{i<j} X^k_{i,j}(n_k - X^k_{i,j}), \frac{1}{n_k} \sum_{i<j} \big(n_{k\oplus} X^k_{i,j} + n_k X^{k\oplus}_{i,j} - 2X^{k\oplus}_{i,j} X^k_{i,j})),$$

applying the independence between both random variable can be expressed as,

$$Cov(D_{G^k}(\mathcal{M}_k), D_{G^{k\oplus}}(\mathcal{M}_k)) = \frac{2}{n_k^2} \sum_{i<j} \big(Cov(X^k_{i,j} n_k, n_{k\oplus} X^k_{i,j}) - 2Cov(X^k_{i,j} n_k, X^{k\oplus}_{i,j} X^k_{i,j}) +$$
$$-Cov((X^k_{i,j})^2, n_{k\oplus} X^k_{i,j}) + 2Cov((X^k_{i,j})^2, X^k_{i,j} X^{k\oplus}_{i,j})).$$

And again each term can be easily expressed in terms of the moments of the binomial distribution.

$\underline{Cov(D_{G^k}(\mathcal{M}_k), D_G(\mathcal{M}_k))}$

$$\mathrm{Cov}(D_{G^k}(\mathcal{M}_k), D_G(\mathcal{M}_k)) = \mathrm{Cov}(D_{G^k}(\mathcal{M}_k), D_{G^k}(\mathcal{M}_k) + D_{G^{k\oplus}}(\mathcal{M}_k))) =$$
$$= \mathrm{Var}(D_{G^k}(\mathcal{M}_k)) + \mathrm{Cov}(D_{G^k}(\mathcal{M}_k), D_{G^{k\oplus}}(\mathcal{M}_k)).$$

The two terms have been previously calculated.

$\underline{\mathrm{Cov}(D_{G^r}(\mathcal{M}_r), D_{G^t}(\mathcal{M}_t)) \quad \text{with} \quad r \neq t}$

$$\mathrm{Cov}(D_{G^r}(\mathcal{M}_r), D_{G^t}(\mathcal{M}_t)) = 0,$$

since $D_{G^r}(\mathcal{M}_r)$ and $D_{G^t}(\mathcal{M}_t)$ are independent random variables

$\underline{\mathrm{Cov}(D_{G^r}(\mathcal{M}_r), D_G(\mathcal{M}_t)) \quad \text{with} \quad r \neq t}$

$$\mathrm{Cov}(D_{G^r}(\mathcal{M}_r), D_G(\mathcal{M}_t)) = \mathrm{Cov}(D_{G^r}(\mathcal{M}_r), D_{G^r}(\mathcal{M}_t) + D_{G^{r\oplus}}(\mathcal{M}_t)) =$$
$$= \mathrm{Cov}(D_{G^r}(\mathcal{M}_r), D_{G^r}(\mathcal{M}_t)) + \mathrm{Cov}(D_{G^r}(\mathcal{M}_r), D_{G^{r\oplus}}(\mathcal{M}_t))$$

Now using that $D_{G^r}(\mathcal{M}_t) = \frac{1}{n_t}\sum_{i<j}\left(n_r X^t_{i,j} + n_t X^r_{i,j} - 2X^r_{i,j}X^t_{i,j}\right)$ we obtain

$$\mathrm{Cov}(D_{G^r}(\mathcal{M}_r), D_{G^r}(\mathcal{M}_t)) = \frac{2}{n_r n_t}\sum_{i<j}\mathrm{Cov}(n_r X^r_{i,j}, n_t X^r_{i,j}) - 2\mathrm{Cov}(n_r X^r_{i,j}, X^r_{i,j}X^t_{i,j}) +$$
$$-\mathrm{Cov}((X^r_{i,j})^2, n_t X^r_{i,j}) + 2\mathrm{Cov}((X^r_{i,j})^2, X^r_{i,j}X^t_{i,j})$$

Since $D_{G^r}(\mathcal{M}_r)$ and $D_{G^{r\oplus}}(\mathcal{M}_t)$ are independent

$$\mathrm{Cov}(D_{G^r}(\mathcal{M}_r), D_{G^{r\oplus}}(\mathcal{M}_t)) = 0$$

.

$\underline{\mathrm{Cov}(D_G(\mathcal{M}_r), D_G(\mathcal{M}_t))}$

$D_G(\mathcal{M}_r) = \sum_{i<j}(n - X^r_{i,j} - X^t_{i,j} - X^{rt\oplus}_{i,j})\frac{X^r_{i,j}}{n_r} + (X^r_{i,j} + X^t_{i,j} + X^{rt\oplus}_{i,j})(1 - \frac{X^r_{i,j}}{n_r})$ and $D_G(\mathcal{M}_t) = \sum_{i<j}(n - X^r_{i,j} - X^t_{i,j} - X^{rt\oplus}_{i,j})\frac{X^t_{i,j}}{n_t} + (X^r_{i,j} + X^t_{i,j} + X^{rt\oplus}_{i,j})(1 - \frac{X^t_{i,j}}{n_t})$

$$\mathrm{Cov}(D_G(\mathcal{M}_r), D_G(\mathcal{M}_t)) = \sum_{i<j}\mathrm{Cov}((n - X^r_{i,j} - X^t_{i,j} - X^{rt\oplus}_{i,j})\frac{X^r_{i,j}}{n_r}, (n - X^r_{i,j} - X^t_{i,j} - X^{rt\oplus}_{i,j})\frac{X^t_{i,j}}{n_t}) +$$
$$+\mathrm{Cov}((n - X^r_{i,j} - X^t_{i,j} - X^{rt\oplus}_{i,j})\frac{X^r_{i,j}}{n_r}, (X^r_{i,j} + X^t_{i,j} + X^{rt\oplus}_{i,j})(1 - \frac{X^t_{i,j}}{n_t})) +$$
$$+\mathrm{Cov}((X^r_{i,j} + X^t_{i,j} + X^{rt\oplus}_{i,j})(1 - \frac{X^r_{i,j}}{n_r}), (n - X^r_{i,j} - X^t_{i,j} - X^{rt\oplus}_{i,j})\frac{X^t_{i,j}}{n_t}) +$$
$$+\mathrm{Cov}((X^r_{i,j} + X^t_{i,j} + X^{rt\oplus}_{i,j})(1 - \frac{X^r_{i,j}}{n_r}), (X^r_{i,j} + X^t_{i,j} + X^{rt\oplus}_{i,j})(1 - \frac{X^t_{i,j}}{n_t}))$$

From here is straighfoward to finish the expression in terms of the moments of the binomial distribution.

$\underline{\mathrm{Cov}(D_G(\mathcal{M}_r), D_{G^t}(\mathcal{M}_t))}$

$$\mathrm{Cov}(D_G(\mathcal{M}_r), D_{G^t}(\mathcal{M}_t)) = \mathrm{Cov}(D_{G^t}(\mathcal{M}_t), D_G(\mathcal{M}_r))$$

The right term was already calculated.

1.4 A1.4- Proof iii: Under H_A

Let write the sample size of each subpopulation as $n_k = c_k n$ where $0 < c_k < 1$, and $\sum_{k=1}^{m} c_k = 1$. The proof is based on the fact that if H_0 *is not true* then for any $d < 0$ there exist a n such that

$$\mathbb{E}(T) < d.$$

Or equivalently,

$$\lim_{n\to\infty} \mathbb{E}(T) = -\infty.$$

$$\mathbb{E}(T) = \sum_{k=1}^{m} \frac{\sqrt{m}}{a} \sum_{k=1}^{m} \sqrt{n_k} \left(\frac{1}{n_k - 1} \mathbb{E}\left(D_{G^k}(\mathcal{M}_k)\right) - \frac{1}{n-1} \mathbb{E}\left(D_G(\mathcal{M}_k)\right) \right) \tag{5}$$

It easy to verify that

$$\mathbb{E}(T) = \frac{\sqrt{m}}{a} \sum_{i<j} \mathbb{E}\left(T^{i,j}\right) := \sum_{i<j} \frac{\sqrt{m}}{a} \sum_{k=1}^{m} \sqrt{n_k} \left(\frac{1}{n_k - 1} \mathbb{E}\left(D^{i,j}_{G^k}(\mathcal{M}_k)\right) - \frac{1}{n-1} \mathbb{E}\left(D^{i,j}_{G}(\mathcal{M}_k)\right) \right), \tag{6}$$

where the sum $\sum_{i<j}$ is over all links, $D^{i,j}_{G^k}(\mathcal{M}_k) = \frac{2}{n_k} X^k_{i,j}(n_k - X^k_{i,j})$ and $D^{i,j}_{G^k}(\mathcal{M}_k) = \frac{2}{n_k} X^k_{i,j}(n_k - X^k_{i,j}) + \frac{1}{n_k}\left(n_{k\oplus} X^k_{i,j} + n_k(\sum_{h\neq k} X^h_{i,j}) - 2(\sum_{h\neq k} X^h_{i,j}) X^k_{i,j}\right)$

For simplicity reasons let suppose that the first $m-1$ groups have a mean network $\tilde{\mathcal{M}}$ with elements $\tilde{\mathcal{M}}(i,j) = p(i,j)$ and the last group m has another mean network $\tilde{\mathcal{M}}_m$ with elements

$$\tilde{\mathcal{M}}_m(i,j) = \begin{cases} p(i,j) \text{ for all } (i,j) \neq (i^*,j^*) \\ q(i,j) \text{ for all } (i,j) = (i^*,j^*), \end{cases}$$

with $q(i^*,j^*) \neq p(i^*,j^*)$. i.e. the mean network differ in only one link. Under this hypothesis,

$$\mathbb{E}(T) = \mathbb{E}\left(T^{i^*,j^*}\right),$$

since the $\mathbb{E}\left(T^{i,j}\right) = 0$ for all $(i,j) \neq (i^*,j^*)$. Now, if we replace $D^{i,j}_{G^k}(\mathcal{M}_k)$ and $D^{i,j}_{G^k}(\mathcal{M}_k)$ and we take expectation it is easy to verify that $\mathbb{E}(T)$ is a quadric expression in $p(i^*,j^*)$ and $q(i^*,j^*)$. If we call $x = p(i^*,j^*)$ and $y = q(i^*,j^*)$, then $\mathbb{E}(T)$ verifies

$$\mathbb{E}(T) = a_1 x^2 + a_2 y^2 + a_3 xy + a_4 x + a_5 y + a_6.$$

Now we now that if $x = y$ (null hypothesis) then $\mathbb{E}(T) = 0$. This means that the there is 1 dimensional subspace that is solution of the equation $\mathbb{E}(T) = 0$. Now, there ara two possibilities for a quadric equation to verifies this last. If there exist another 1 dimensional space for the equation $\mathbb{E}(T) = 0$ then the function $\mathbb{E}(T)$ is an hyperbolic paraboloid, if not the function $\mathbb{E}(T)$ is a parabolic cylinder. In order to distinguish between these two cases we will move a little ($\epsilon << 1$) to both sides of the found solution for $\mathbb{E}(T) = 0$ (the line x=y) and see if the sign of $\mathbb{E}(T)$ change. If the sign changes then $\mathbb{E}(T)$ is an hyperbolic paraboloid, if not $\mathbb{E}(T)$ is a parabolic cylinder.

We will study $\mathbb{E}(T)$ for $(x_1, y_1) = (1/2, 1/2 + \epsilon)$ and for $(x_2, y_2) = (1/2, 1/2 - \epsilon)$ with $\epsilon > 0$. For simplicity we will study $\lim_{n\to\infty} \frac{1}{\sqrt{n}} \mathbb{E}(T)$ which is enough for proof [1].

It is straightforward to see that for both (x_1, y_1) and (x_2, y_2)

$$\lim_{n\to\infty} \frac{1}{\sqrt{n}} \mathbb{E}(T) = -2(1 - c_m)\sqrt{c_m}\epsilon^2,$$

1 Based on T it is easy to see that the rate of convergence $\frac{1}{\sqrt{n}}$

which is negative value since $0 < c_m < 1$, confirming that $\mathbb{E}(T)$ is a parabolic cylinder that goes than, i.e. if $q(i^*, j^*) \neq p(i^*, j^*)$ then

$$\lim_{n \to \infty} \mathbb{E}(T) = -\infty.$$

To finish the proof we say that any other alternative hypothesis can be proved from this particular alternative scenario. For example, if there exist another (i^{**}, j^{**}) with $p(i^{**}, j^{**}) \neq q(i^{**}, j^{**})$ then

$$\mathbb{E}(T) = \mathbb{E}\left(T^{i^*, j^*}\right) + \mathbb{E}\left(T^{i^{**}, j^{**}}\right)$$

and we apply the same proof for each term. Another alternative hypothesis might be that there exist a unique (i^*, j^*) where $p_r(i^*, j^*) \neq p_s(i^*, j^*)$ for $r \neq s$ being $p_r(i^*, j^*)$ the probability of observing link (i^*, j^*) in subpopulation r. In this case $\mathbb{E}(T)$ is a quadric expression in $p_1(i^*, j^*)$, $p_2(i^*, j^*)$, ..., and $p_m(i^*, j^*)$. And the same argument can be used obtaining the same result, under the alternative hypothesis $\lim_{n \to \infty} \mathbb{E}(T) = -\infty$.

2 A2- EXAMPLE 1

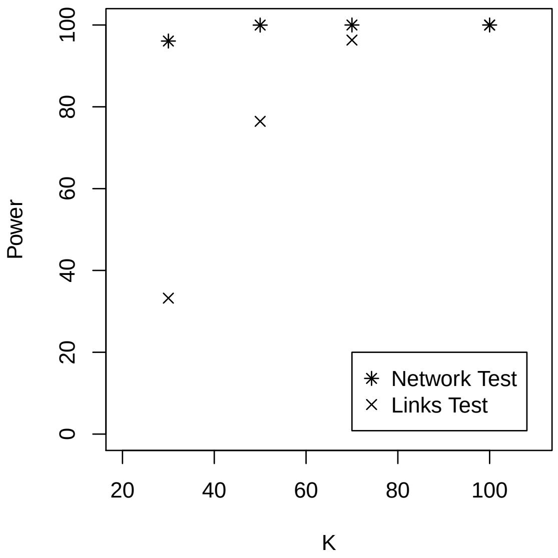

Figure S1: Fig A1: Power of the tests as a function of the sample size for the model with parameters $\lambda_1 = 0.5$, $\lambda_2 = 2/3$, and $\lambda_3 = 0.5$.

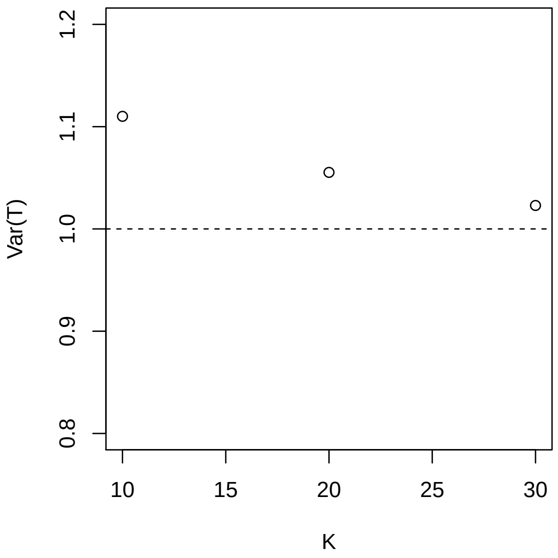

Figure S2: Fig A2: **Null hypothesis.** Variance of T statistics as a function of the sample size, K for the model with parameters $\lambda_1 = 0.5$, $\lambda_2 = 0.8$, and $\lambda_3 = 0.6$.

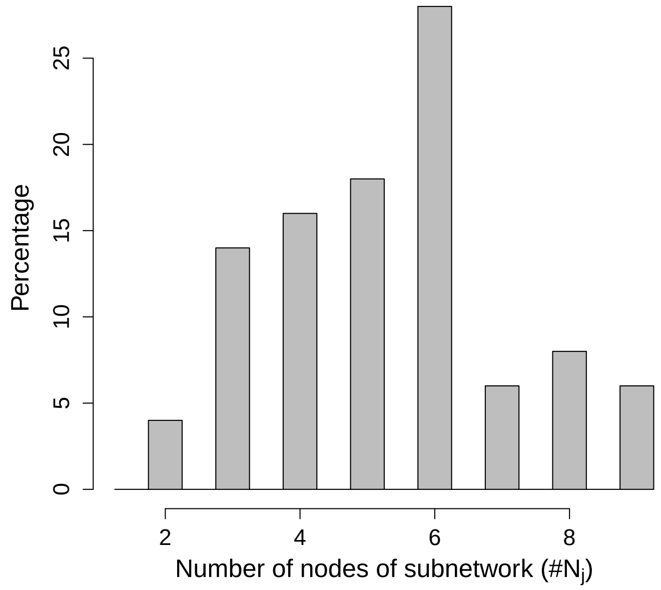

Figure S3: Fig A3: Histogram of number of nodes determine by identification procedure. These results correspond to the model with parameters $\lambda_1 = 0.5$, $\lambda_2 = 0.8$, $\lambda_3 = 0.6$ and $K = 30$.

3 A3- HCP RESTING-STATE FMRI FUNCTIONAL NETWORKS

For each variable, we calculated W_3 as well as W_4 and W_5, which counts the number of T statistics lower than -4 and -5, respectively. Using a resampled bootstrap, we obtained empirical probabilities $P(W_5 \geqslant 1) = 0$ (less than 1/10000) and $P(W_4 = 1) = 1/10000$. Variables with W_3 values greater than 3 are shown in Table S1 and S2.

Brain volumetric variable	W_3	W_4	W_5
FS–R–Inferiortemporal–Area	5	5	3
FS–SupraTentorial–Vol	5	5	3
FS–R–WM–Vol	5	4	3
FS–R–Cort–GM–Vol	6	3	3
FS–BrainSeg–Vol	5	3	3
FS–Tot–WM–Vol	5	3	3
FS–Mask–Vol	5	3	3
FS–L–Middletemporal–Area	9	4	2
FS–R–Cuneus–Area	5	4	2
FS–L–Lateraloccipital–Area	4	4	2
FS–R–Superiorfrontal–Area	5	3	2
FS–BrainSeg–Vol–No–Vent	5	3	2
FS–L–Supramarginal–Area	5	3	1
FS–R–Fusiform–Area	5	2	2
FS–BrainStem–Vol	5	2	1
FS–R–Precentral–Area	5	2	1
FS–L–Superiorfrontal–Area	4	3	2
FS–L–WM–Vol	4	3	2
FS–OpticChiasm–Vol	4	2	2
FS–R–Rostralmiddlefrontal–Area	4	2	1

Table S1: Variables that partitioned the subjects in groups that present very high statistical differences between the corresponding brain networks. Only variables with $W_3 \geqslant 4$ are included.

Behavioral variables (label)	W_3	W_4	W_5
Amount of sleep (PSQI_AmtSleep)	9/8/7	6/6/7	4/6/6
Cognitive flexibility (CardSort_AgeAdj)	4/3/4	3/3/4	0/3/3
Cognitive flexibility (CardSort_Unadj)	4/3/3	0/2/2	0/2/2
Motor (Strength_AgeAdj)	4/3/3	4/3/3	3/2/2
Motor (Strength_Unadj)	4/3/4	4/2/3	2/2/2
Working memory (WM_Task_2bk_Acc)	3/5/1	0/2/1	0/0/1
Relational processing (Relational_Task_Acc)	3/4/7	0/3/6	0/0/5
Delay discounting (DDisc_SV_10yr_40K)	1/5/7	0/1/6	0/0/6
Delay discounting (DDisc_AUC_40K)	0/4/4	0/3/4	0/3/4

Table S2: Behavioral variables that partitioned the subjects in groups that present high statistical differences between the corresponding brain networks. The W_3, W_4 and W_5 statistics are presented for different networks sizes (15 / 50 / 300). Only variables with $W_3 \geqslant 4$ are included.

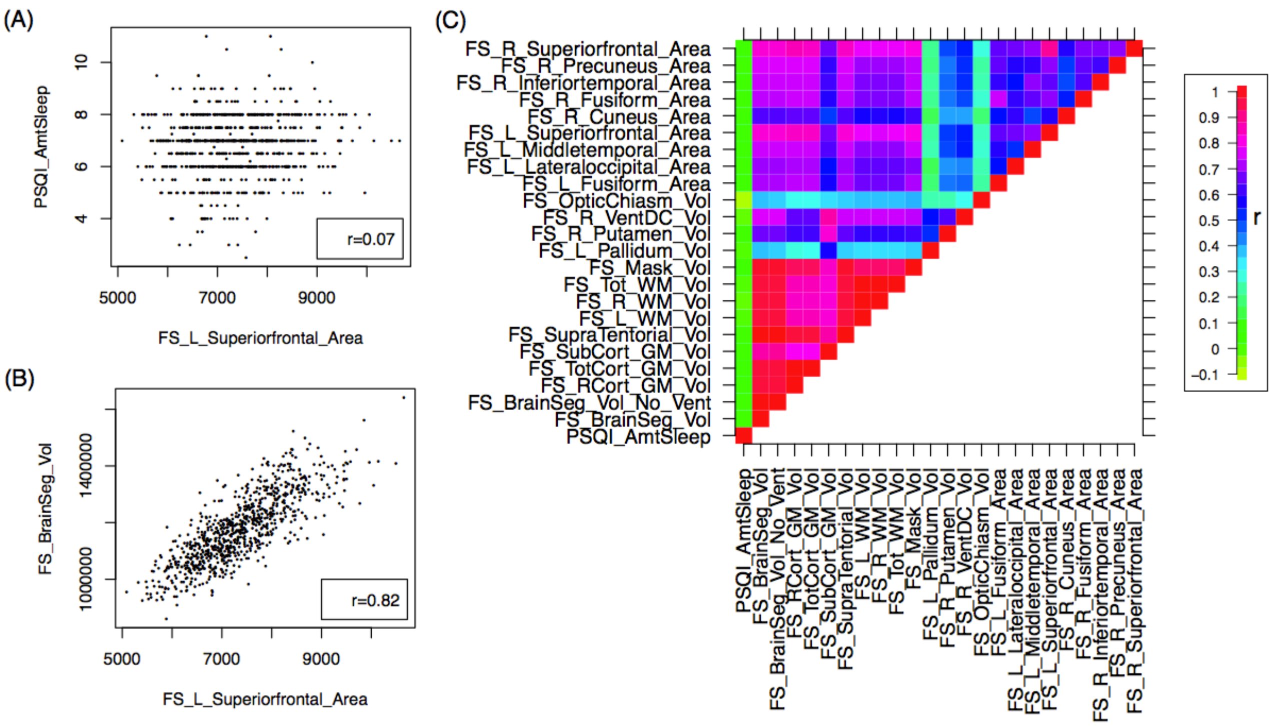

Figure S4: Fig A4: Relationship between variable Right Inferiortemporal Area and variable: (A) Amount of sleep , (B) Brain segmentation volume. The Spearman correlation coefficient between both variables are shown. (C) Spearman correlation matrix between the highly significant variables.

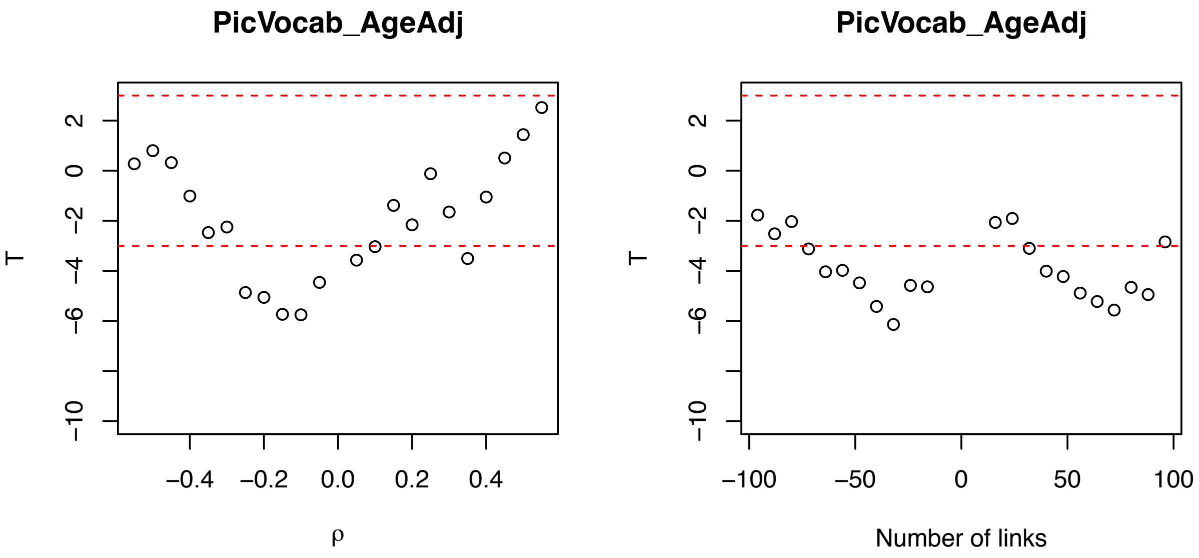

Figure S5: Fig A5: T–statistics as a function of (left panel) ρ and (right panel) the number of links for the variable *Picture vocabulary test*.
